# Study on Expansion Rate of Steel Slag Cement-Stabilized Macadam Based on BP Neural Network

**DOI:** 10.3390/ma17143558

**Published:** 2024-07-18

**Authors:** Hengyu Wu, Feng Xu, Bingyang Li, Qiju Gao

**Affiliations:** School of Civil Engineering, Suzhou University of Science and Technology, Suzhou 215009, China; mulberry0108@gmail.com (H.W.);

**Keywords:** steel slag, cement-stabilized macadam, road base, neural network, expansion rate

## Abstract

The physicochemical properties of steel slag were investigated using SEM and IR, and it was found that free calcium oxide and free magnesium oxide in steel slag produce calcium hydroxide when in contact with water, leading to volume expansion. Thus, the expansion rate of steel slag itself was first investigated, and it was found that the volume expansion of steel slag was more obvious in seven days after water immersion. Then, the cement dosages of 5% and 6% of the steel slag expansion rate and cement-stabilized gravel volume changes between the intrinsic link were further explored after the study found that the cement bonding effect can be partially inhibited due to the volume of expansion caused by the steel slag, so it can be seen that increasing the dosage of cement can reduce the volume expansion of steel slag cement-stabilized gravel with the same dosage of steel slag. Finally, a prediction model of the expansion rate of steel slag cement-stabilized gravel based on the BP (back propagation) neural network was established, which was verified to be a reliable basis for predicting the expansion rate of steel slag cement-stabilized aggregates and improving the accuracy of the proportioning design.

## 1. Introduction

Cement-stabilized crushed stone uses graded crushed stone as aggregate, fills the voids between aggregates with a certain amount of binder material and sufficient mortar volume, and compacts it [1]. Its compactness approaches density, and its strength mainly relies on the principle of interlocking between crushed stones, while having enough mortar volume to fill the voids of the aggregate. It has a high initial strength and rapidly increasing strength with age, forming a slab structure, thus possessing higher strength, as well as good resistance to permeability and freezing [2].

Ma et al. [3] added polypropylene long fiber to cement-stabilized crushed stone and observed, through scanning electron microscopy, that its flexural fatigue performance was significantly enhanced. Yang et al. [4] successfully improved the compressive strength and frost resistance by adding rubber particles into cement-stabilized crushed stone. Li et al. [5] found that the addition of basalt fibers into cement-stabilized crushed stone resulted in varying degrees of improvement in durability and mechanical properties. Liu et al. [6] validated the feasibility of using aeolian sand to replace fine aggregate in subbase materials, concluding that 100% substitution of aeolian sand for fine aggregate is entirely feasible in terms of strength and corrosion resistance. Considering the increasing industrial emissions of phosphogypsum, Sun et al. [7] proposed using phosphogypsum to replace the graded crushed stone in the preparation of cement-stabilized crushed stone. Experimental results showed that phosphogypsum subgrade materials could meet the strength requirements, and leaching experiments verified that phosphogypsum would not leach out and cause environmental pollution. Zhao et al. [8] found that the addition of a fine powder adversely affected the crack resistance and dewatering performance of cement-stabilized crushed stone through their research. Zeng et al. [9] prepared semi-rigid pavement base layers using coral reef limestone crushed stone as the main material, showing promising application prospects in marine engineering. Hoy et al. [10] discovered that the addition of natural latex improved the durability of cement-stabilized crushed stone against dry–wet cycles. Yan et al. [11] utilized coal solid waste to prepare cement-stabilized crushed stone, verifying its enormous potential as a new environmentally friendly pavement base material through mechanical property tests and carbon emission assessments.

Steel slag is a by-product of the steel manufacturing industry, primarily produced in electric arc furnaces, consisting of iron, aluminum, calcium oxide, and magnesium oxide [12]. After solid waste is generated during processes such as ironmaking, steel slag can be obtained by removing impurities from molten steel using filters. Li et al. [13] showed that steel slag has a lower crushing value, as compared to other natural aggregates, and Marina et al. [14] illustrated the same pattern for the Los Angeles abrasion value of steel slag, which proves that steel slag will have better strength and durability when applied in road base. The study by Ren et al. [15] mentioned that steel slag is 10–50% denser than natural aggregates, so it is important to focus on the cheap price of steel slag while also weighing its transportation costs. Chen et al. [16] observed the pore size distribution of cement-stabilized crushed stone after adding steel slag through nuclear magnetic resonance, revealing that steel slag, due to its good locking effect, micro-aggregate effect, and volcanic ash effect, can significantly improve pore size distribution and reduce the content of large pores. Li et al. [17] studied the drying shrinkage and temperature shrinkage characteristics of steel slag cement-stabilized crushed stone, successfully controlling the shrinkage strain caused by temperature by adjusting the gradation. Lang et al. [18] tested the relationship between the unconfined compressive strength of steel slag powder and cement-stabilized crushed stone, finding that the strength initially increased and then decreased with the increase in steel slag content.

Artificial neural networks (ANNs) are a nonlinear data modeling method inspired by the simulation of biological neural networks for information processing [19]. Generally, artificial neural networks consist of three parts: an input layer, output layer, and hidden layer. Information is transmitted and analyzed through neurons, and after weighted processing, a nonlinear result can be output [20]. Nowadays, the application of neural networks for prediction has become widespread in cement-stabilized crushed stone research [21,22,23,24,25].

Steel slag, containing a significant amount of f-CaO and f-MgO, reacts extensively with free water to produce Ca(OH)_2_, resulting in volumetrically unstable expansion [26], thus causing expansion in cement-stabilized crushed stone. However, there is limited research on the expansiveness of steel slag cement-stabilized crushed stone. Therefore, this study conducted research on the expansion rates of steel slag and steel slag cement-stabilized crushed stone and established a BP (back propagation) neural network prediction model to validate its feasibility in predicting the expansion rate of cement-stabilized crushed stone.

## 2. Experimental Materials

### 2.1. Cement

Hai Luo brand P·O 42.5 ordinary Portland cement (OPC) was used in the experiments. The specific environment in this experiment belongs to an indoor dry environment. According to the EN 206 standard [27], the external exposure class of this environment is XC1. The performance indicators of the cement are shown in Table 1.

### 2.2. Aggregates

**Figure 1:**
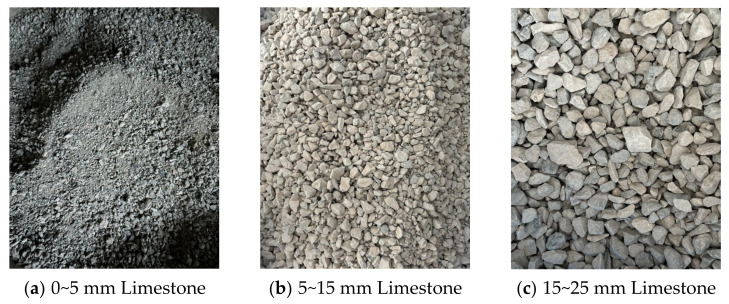
Limestone crushed stone of various particle sizes.

The experiment selected aggregates with three particle size ranges: 0~5 mm, 5~15 mm, and 15~25 mm, as shown in Figure 1.

### 2.3. Steel Slag

**Figure 2:**
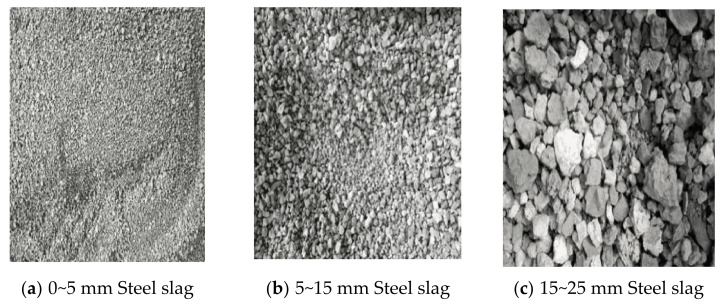
Steel slag of various particle sizes.

The steel slag used in this experiment was produced by Baosteel Group Corporation Limited in Shanghai and is basic oxygen furnace (BOF) slag. The slag used in the experiment had been stored for 12 months after production, with particle sizes of 0~5 mm, 5~15 mm, and 15~25 mm, corresponding to the limestone particle sizes mentioned above, as shown in Figure 2.

#### 2.3.1. The Physical Properties of Steel Slag

The physical properties of steel slag are shown in Table 2.

#### 2.3.2. The Chemical Properties of Steel Slag

**Figure 3:**
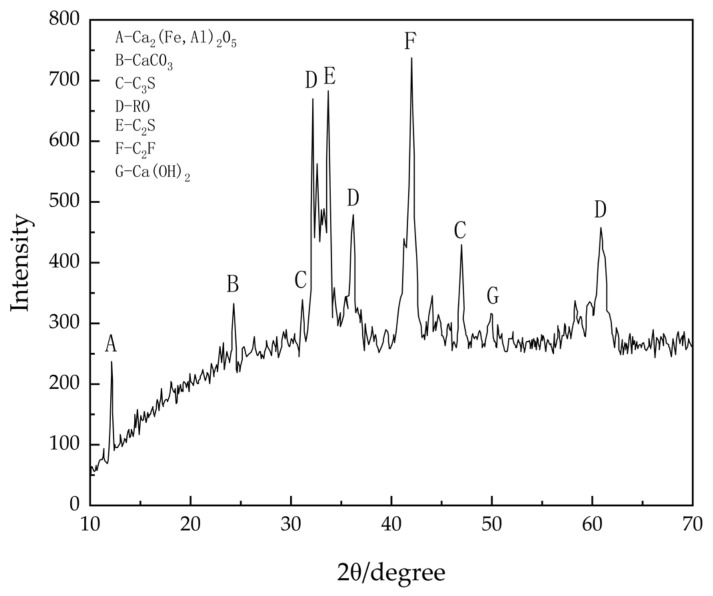
Steel slag XRD spectrum.

The XRD results are shown in Figure 3, which indicated that the main chemical components present in steel slag include iron-aluminum-calcium, calcium hydroxide, calcium carbonate, tricalcium silicate, as well as some phases of RO, dicalcium silicate, and free calcium oxide. Further examination of the spectra revealed the presence of numerous peaks, indicating the presence of various minerals in the steel slag.

During the process of daily stacking, steel slag produces calcium hydroxide, which then reacts with carbon dioxide gas to form calcium carbonate. This explains the detection of calcium carbonate in the spectra. Additionally, it was observed from the spectra that steel slag contains substances similar to those found in cement, such as C_3_S and C_2_S. These substances undergo hydration and secondary hydration reactions, imparting certain cementitious properties to the steel slag and enhancing its mechanical performance.

#### 2.3.3. Microstructure of Steel Slag

**Figure 4:**
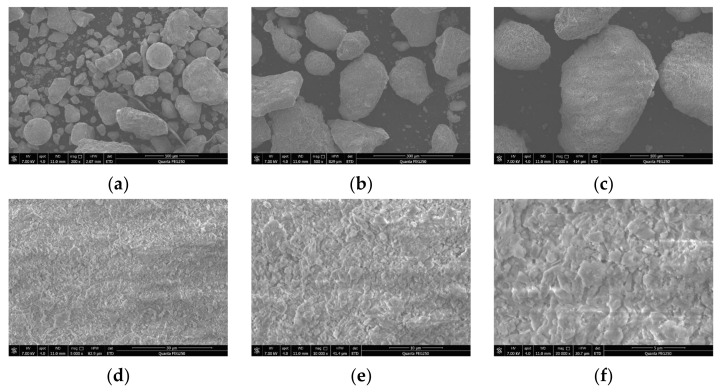
The microstructural characteristics of steel slag at different magnifications. (**a**) Sample magnified at 200 times, (**b**) sample magnified at 500 times, (**c**) sample magnified at 1000 times, (**d**) sample magnified at 5000 times, (**e**) sample magnified at 10,000 times, and (**f**) sample magnified at 20,000 times.

Observation of the morphology of steel slag was conducted using a scanning electron microscope (FEI Quanta FEG250). Steel slag samples were observed at magnifications of 200 times, 500 times, 1000 times, 5000 times, 10,000 times, and 20,000 times to examine the morphological characteristics of steel slag at different magnifications, as shown in Figure 4.

As can be seen from Figure 4a,b, in the case of low-magnification observation of the sample at 200 and 500 times, the overall appearance of the steel slag particles roughly presented an elliptical shape, and the steel slag particles were less angular compared to ordinary crushed stone.

From the Figure 4c,d observations, with further magnification, the roughness of the surface of the steel slag began to be seen on the graph, and it can be intuitively found that many tiny pores began to appear on its surface.

From Figure 4e,f, when the magnification reached 10,000 and 20,000 times, a lot of pores could be clearly seen, which may be the reason that the water absorption rate of steel slag is much larger than that of gravel.

#### 2.3.4. Comprehensive Thermal Analysis of Steel Slag

**Figure 5:**
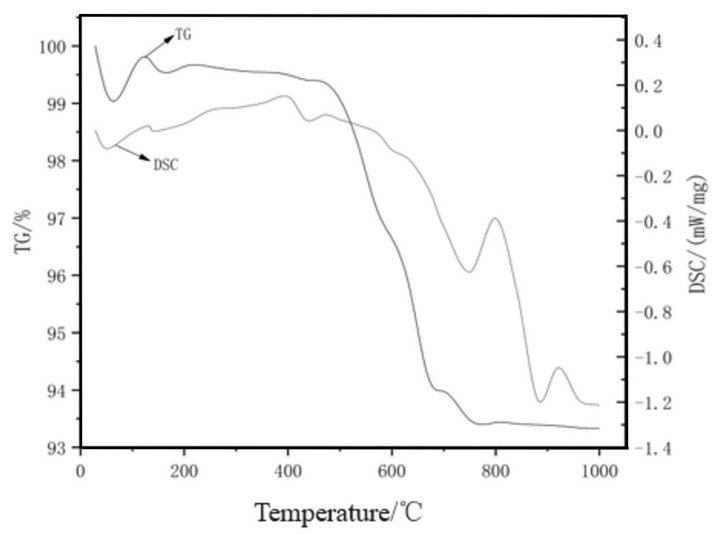
TG-DSC curve of steel slag.

To measure the mass change and heat flow change of steel slag at different temperatures, a NETZSCH 449F3 Synchronous Thermal Analyzer was used in the test. The temperature range was set from 40 to 1000 °C, with a heating rate of 10 °C/min. The results are shown in Figure 5.

Observing the DSC curve in the figure, multiple endothermic valleys were found within the test temperature range of 40–1000 °C. At around 50 °C, a smaller endothermic valley appeared, and the TG curve initially decreased and then increased. This phenomenon is likely due to the carbonation reaction of the steel slag. Within the test temperature range of 95–350 °C, an endothermic valley caused by the vaporization of free water and bound water was observed. In the temperature range of 350–500 °C, another endothermic valley appeared, and the TG curve showed a significant decrease. This is mainly due to the endothermic decomposition of some uncarbonated Ca(OH)_2_ in the steel slag, resulting in the formation of calcium oxide and water, which led to a mass reduction. Between 500 °C and 800 °C, the TG curve continued to decline. Within the temperature range of 700–800 °C, the DSC curve showed a distinct endothermic trough, which is attributed to the decomposition of the RO phase and other minerals. When the temperature reached 800–1000 °C, the TG curve decreased again due to the decomposition of CaCO_3_. From the results of the comprehensive thermal analysis test, it can be seen that the properties of steel slag can remain stable at a drying temperature of around 100 °C. Therefore, steel slag can exhibit good thermal stability when used in subgrade engineering applications.

#### 2.3.5. Testing of Steel Slag for Water Swelling by Immersion

In the hydration reaction, free calcium oxide and magnesium oxide produced primarily amorphous and small crystalline Ca(OH)_2_. As the hydration reaction progressed, these amorphous and small crystalline forms of Ca(OH)_2_ underwent further crystallization. From the results of the study, it was found that, as crystallization progressed, the volume of Ca(OH)_2_ crystals and the expansion of steel slag increased accordingly, leading to the expansion of steel slag. Table 3 shows the swelling values of steel slag after hydration reaction occurs when it is immersed in water.

**Figure 6:**
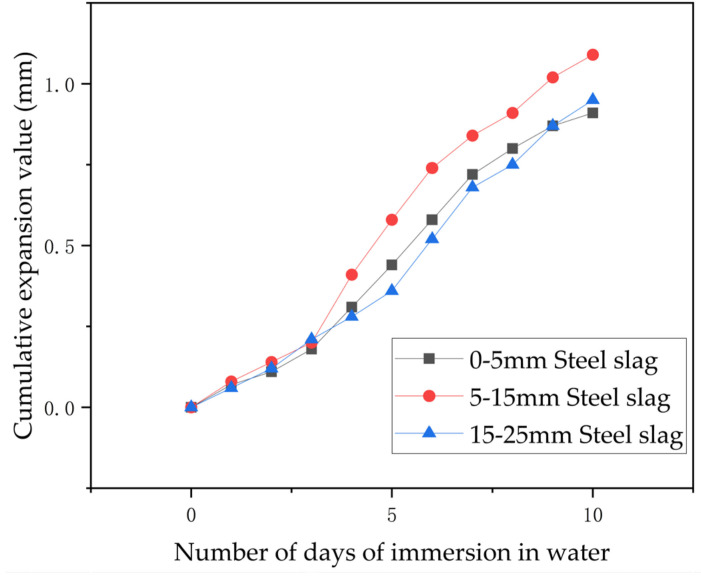
Cumulative expansion values of steel slag of various grain sizes by immersion as a function of time.

From Figure 6, it is evident that the cumulative expansion of steel slag increased with the immersion time for each grain size. Initially, over the first three days, this expansion showed a nearly linear progression. Subsequently, after the third day, the rate of cumulative expansion accelerated, slowing down again after the seventh day. Overall, the cumulative expansion for each particle size steadily increased with longer immersion times, with a noticeable slowing in the rate of increase beyond the seventh day.

## 3. Grading and Proportioning of Steel Slag Cement Stabilized Macadam

**Figure 7:**
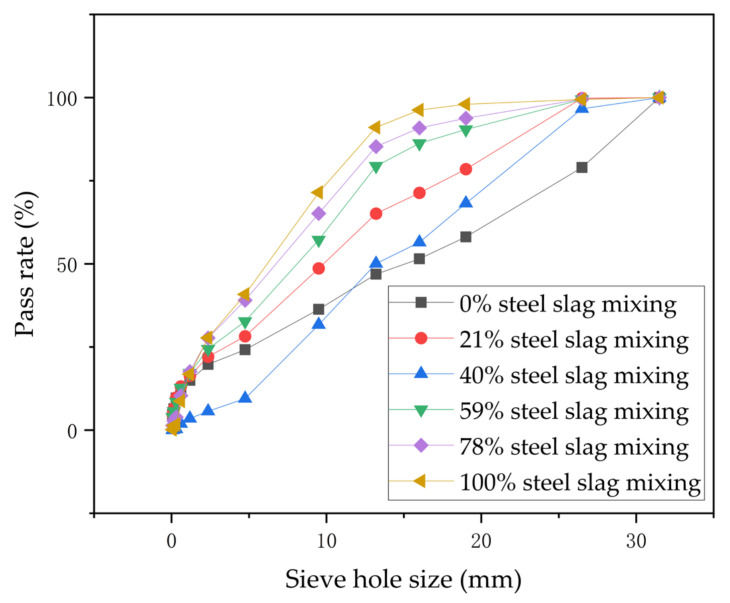
Synthetic grading curves for steel slag cement-stabilized macadam mixes.

Six steel slag substitution rates of 0%, 21%, 40%, 59%, 78%, and 100% were selected, and the specific ratios are shown in Table 4. The corresponding grades were fitted to the curves, as shown in Figure 7.

## 4. Research on the Expansion Rate of Steel Slag-Stabilized Crushed Stone Mixture Based on BP Neural Network

### 4.1. The Natural Expansion Rate of the Mixture

Standard specimens with six different steel slag contents were prepared: 0%, 21%, 40%, 59%, 78%, and 100%, with cement contents of 5% and 6%. The specimens were divided into six equal sections along the height, and each section was measured using a vernier caliper. The diameter was divided into three equal sections according to angles and measured at each section. After curing for seven days, the measurements were repeated using the same method to calculate the volume of the specimens after curing. The volume expansion of the mixture in its natural state when 5% and 6% cement was added is shown in Table 5 and Table 6.

**Figure 8:**
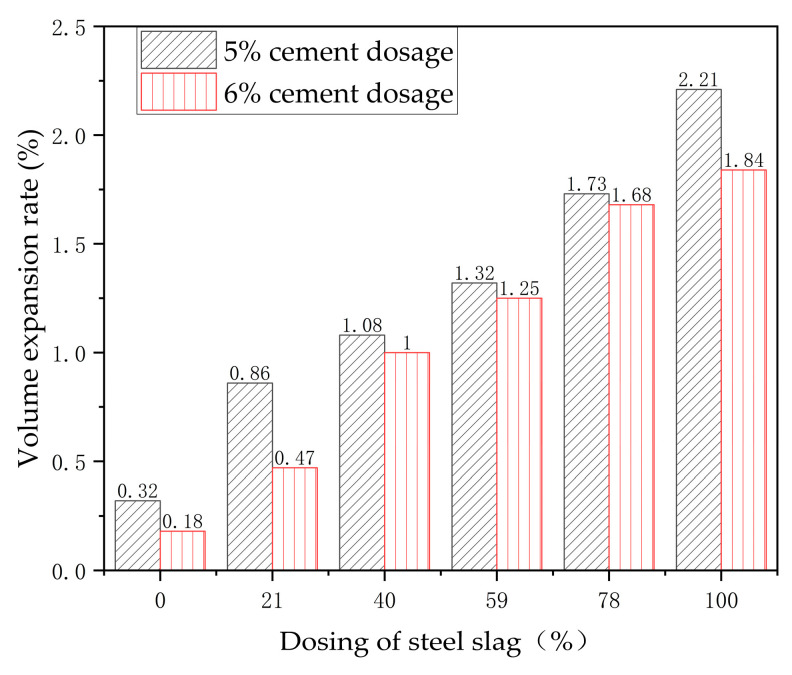
Results of the expansion test in natural state.

From Figure 8, it is observed that, on one hand, with the increasing dosage of cement additives, the overall volume expansion rate of the mixture decreased. On the other hand, as the amount of steel slag added gradually increased, the volume expansion rate of the mixture also increased accordingly. However, when the slag content reached 78%, the overall volume expansion rate exceeded 1.5%, which is not suitable for engineering practice.

### 4.2. The Immersion Expansion Rate of the Mixture

In the cases where 5% and 6% cement were used, with the volume expansion rate of the mixture without steel slag as the baseline, experiments were conducted to determine the immersion expansion rate after sequentially replacing the mixture with steel slag contents of 21%, 40%, 59%, 78%, and 100%. The results are shown in Table 7 and Table 8.

Roughly speaking, from Table 7 and Table 8, it can be observed that with the increase in steel slag content, the expansion rate of the mixture gradually increased. Additionally, with the increase in immersion days, the expansion rate of the mixture also gradually increased. Furthermore, comparing Table 7 and Table 8, it is noted that as the cement content increased, the expansion rate of the mixture under the same steel slag content and immersion time decreased. This is mainly due to the increase in cement dosage, which made the structure of the mixture denser, making it more difficult for moisture to penetrate the specimen. Moreover, with the increase in the bonding effect, it also offset some of the expansion of the steel slag.

**Figure 9:**
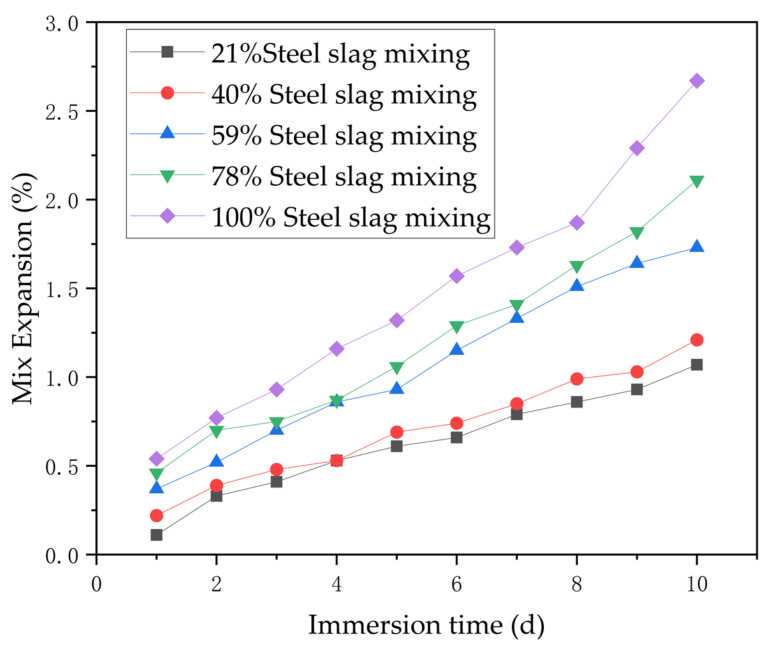
The immersion expansion rate of the mixture when 5% cement was added.

**Figure 10:**
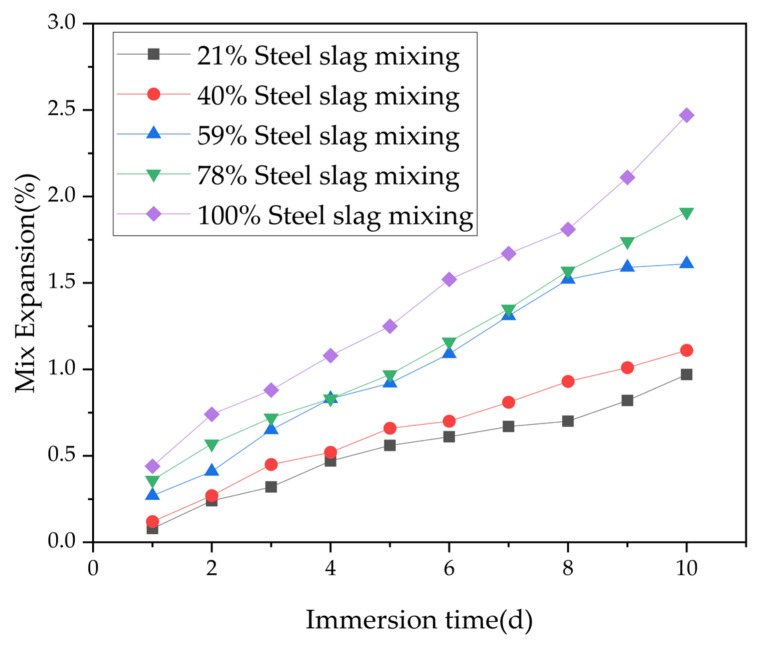
The immersion expansion rate of the mixture when 6% cement was added.

According to the experimental results presented earlier, the immersion expansion rate curve of steel slag itself followed the same trend as the expansion rate curve of the steel slag cement-stabilized crushed stone mixture, as shown in Figure 9 and Figure 10.

The expansion changes in the steel slag mixture after immersion are mainly attributed to the contact between steel slag and water. The reactive substances contained in steel slag react with water, resulting in expansion and causing the mixture to expand accordingly. Therefore, under the same immersion time conditions, there is an inherent connection between the overall volume expansion changes of the steel slag mixture and the expansion of the steel slag material itself.

### 4.3. Construction and Test of Expansion Volume Prediction Model Based on BP Neural Network

The model data-processing steps involved setting training sample data and prediction data, normalizing training sample data, setting training parameters, and constructing the BP neural network, including training, neural network prediction, back-normalization of prediction results, and error calculation. The accuracy of the model prediction results is commonly used to evaluate the mean square error, sample correlation coefficient, and other indicators.

#### 4.3.1. Selection of Training and Test Samples

One group of each steel slag dosage out of fifty groups of immersion expansion rate change values of mixes with 5% cement was taken as the test sample, and the remaining forty-five groups were taken as training samples. The training and test samples are shown in Table 9 and Table 10.

#### 4.3.2. Prediction Results Output

From Table 11, it can be found that the difference between the values produced after the prediction of the model constructed by the neural network and the measured values was less than 0.1%, which is a small error, so the model can be applied to the prediction of the relevant performance.

**Figure 11:**
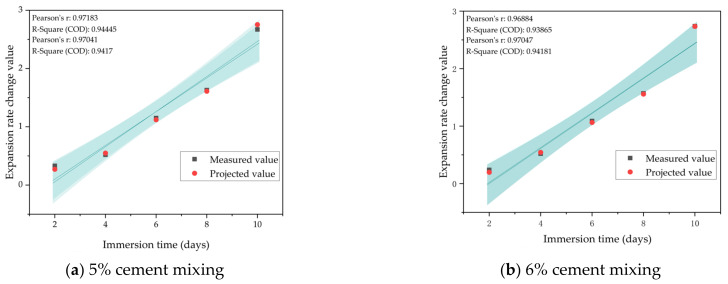
Fitting results of the predicted and actual values of the expansion rate of the mixture at 5% and 6% cement content.

The BP neural network numerical prediction of the expansion rate of the mixture under 6% cement content was carried out through the same step, and the fitting results of the predicted and actual values of the mixture expansion rate under 5% and 6% cement content are shown in Figure 11.

**Figure 12:**
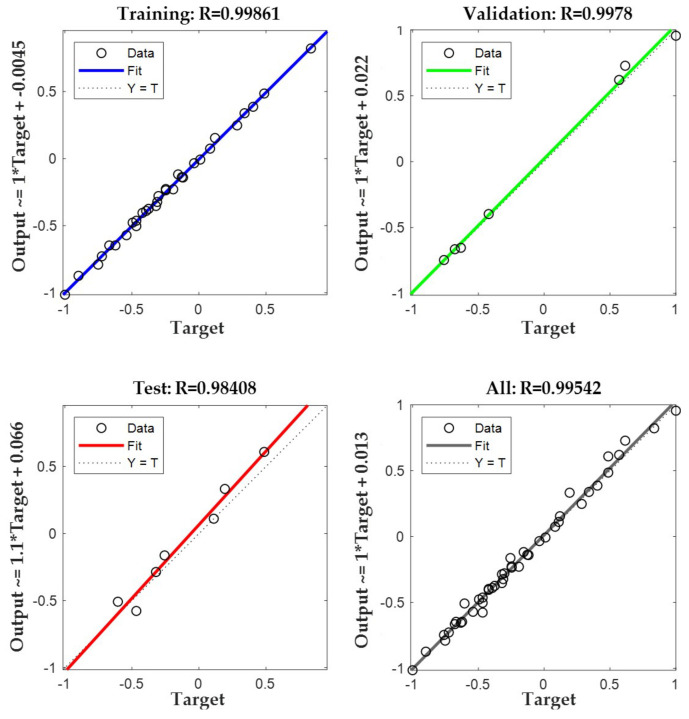
Correlation coefficients of the BP model for stable gravel samples mixed with 5% cement.

**Figure 13:**
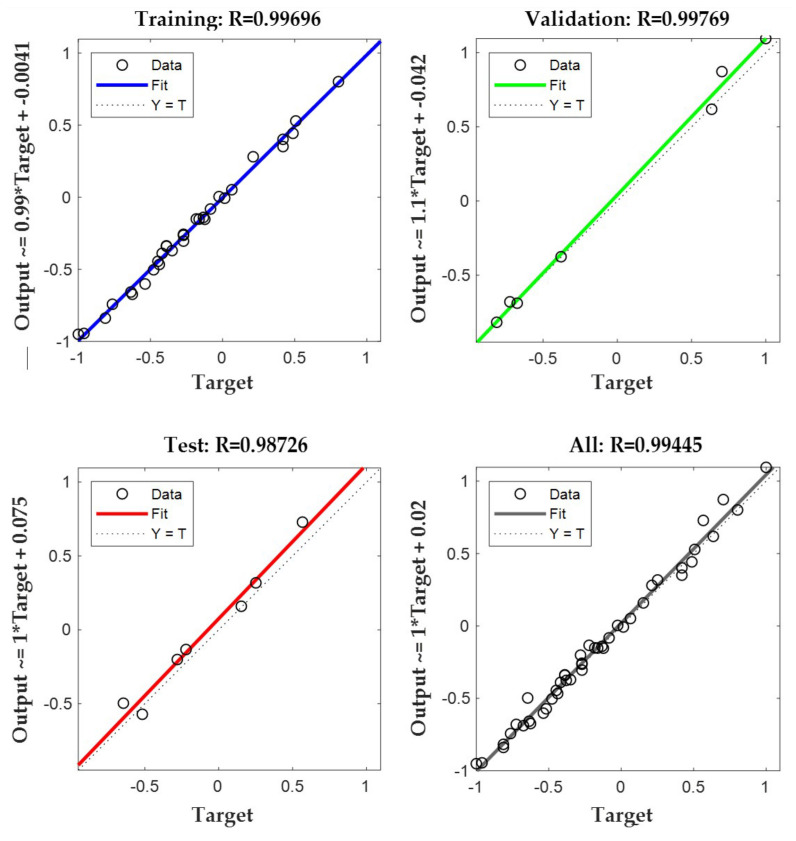
Correlation coefficients of the BP model for stable gravel samples mixed with 6% cement.

In the prediction with the BP neural network, the more the correlation coefficient of the samples converged to 1, the more it indicated that the results of the training of the model were satisfactory. After the model training, the correlation results were as shown in Figure 12 and Figure 13.

As can be seen from the figures, the correlation coefficients (R) of each sample of the mixtures at 5% and 6% cement mixing were more than 0.9, in which the correlation coefficients of the training samples at 5% cement mixing reached 0.99861, and the correlation coefficients of the training samples at 6% cement mixing reached 0.99769. The combination of the various indexes in Table 12 showed that the neural network model used in this paper has a good training effect and the credibility of its model is high.

## 5. Conclusions

1. It was found that the volume expansion rate of cement-stabilized gravel mixtures with different steel slag dosages over seven days, in the case of cement dosages of 5% and 6%, in the natural state increased with the gradual increase of steel slag substitution, and when the dosage reached 59%, it was no longer suitable for use in actual projects.
2. The rule of change of the volumetric expansion rate of the mixture in the submerged state was consistent with the natural state. In the same amount of steel slag and with the same submersion time, with the increase in the amount of cement, the expansion rate of the mixture decreased, which was mainly due to the cement’s bonding effect being enhanced, offsetting part of the expansion caused by the steel slag.
3. The expansion rate polynomial of the cement-stabilized steel slag crushed stone mix was fitted using MATLAB 2020b software, and a BP neural network prediction model of the expansion rate of the cement-stabilized steel slag crushed stone mix was established. This method roughly arrived at the expansion rate of cement-stabilized crushed stone steel slag mix through the calculation of the expansion rate of steel slag and the different substitution amounts of steel slag. It greatly reduced the experimental workload, improved the accuracy of the proportion design, and provided a reliable method for the prediction and quality control of the expansion of the cement-stabilized steel slag aggregate.
4. The expansion damage of steel slag is an important factor affecting the application of steel slag cement-stabilized macadam, and its expansion is a long-term process. It is recommended to conduct long-term research and attempt to incorporate other cost-effective materials to further explore ways to effectively utilize the excellent performance of steel slag while economically addressing its drawbacks.

## Figures and Tables

**Table 1:** Cement test results.

Indicators		Units	Technical Standards	Measured Values
Specific surface area		m^2^/kg	≥300	344
Stability		mm	≤5.0	1.8
Initial setting time		min	≥45	183
Final setting time		min	≤600	244
Loss on burning		%	≤5.0	3.98
Sulfur trioxide		%	≤3.5	2.07
Magnesium oxide		%	≤5.0	2.64
Chloride ion		%	≤0.06	0.007
3-day strength	Flexural strength	MPa	≥3.5	5.7
	Compressive strength	MPa	≥17.0	28.0
28-day strength	Flexural strength	MPa	≥6.5	8.9
	Compressive strength	MPa	≥42.5	50.7

**Table 2:** Steel slag performance test results.

Physical Properties	0~5 mm	5~15 mm	15~25 mm	Technical Requirement
Apparent relative density (g/cm^3^)	3.664	3.755	3.549	≥2.6
Packing density (g/cm^3^)	2.352	2.185	1.941	/
Gross volume relative density (g/cm^3^)	3.422	3.572	3.827	/
Void ratio (%)	35.8	41.8	45.3	/
Water absorption (%)	2.03	1.36	1.89	≤2
Sand equivalent (%)	71	/	/	/
Crushing value (%)	/	16.1	17.6	≤22
Los Angeles abrasion value (%)	/	16.4	17.2	≤26
Needle flake content (%)	/	2.2	2.7	≤22

**Table 3:** Test values of steel slag specimens for water swelling by immersion.

Days		D0	D1	D2	D3	D4	D5	D6	D7	D8	D9	D10	Expansion Rate
A	Before heating	0	0.07	0.11	0.18	0.31	0.44	0.58	0.72	0.80	0.87	0.91	0.76%
	After heating	0.04	0.10	0.15	0.24	0.37	0.49	0.61	0.77	0.85	0.89		
B	Before heating	0	0.08	0.14	0.20	0.41	0.58	0.74	0.84	0.91	1.02	1.09	0.91%
	After heating	0.03	0.12	0.18	0.27	0.43	0.61	0.78	0.89	0.93	1.05		
C	Before heating	0	0.06	0.12	0.21	0.28	0.36	0.52	0.68	0.75	0.87	0.95	0.79%
	After heating	0.04	0.11	0.17	0.26	0.32	0.44	0.56	0.71	0.79	0.90		

(A is 0~5 mm steel slag, B is 5~15 mm steel slag, and C is 15~25 mm steel slag).

**Table 4:** The aggregate composition ratios under each steel slag blending amount.

Serial Number	Gathering Gear					
	15~25 mm Limestone	5~15 mm Limestone	0~5 mm Limestone	15~25 mm Steel Slag	5~15 mm Steel Slag	0~5 mm Steel Slag
1	37%	32%	31%	0%	0%	0%
2	31%	21%	21%	13%	10%	4%
3	20%	13%	19%	25%	15%	8%
4	10%	8%	16%	37%	17%	12%
5	5%	7%	5%	31%	20%	32%
6	0%	0%	0%	32%	31%	37%

**Table 5:** The volume expansion of the mixture in its natural state when 5% cement was added.

Type of Mix	Initial Volume (cm^3^)	Volume after 7 Days (cm^3^)	Volume Expansion Rate (%)
0% steel slag mixing	2654.89	2663.39	0.32
21% steel slag mixing	2675.12	2698.13	0.86
40% steel slag mixing	2648.11	2676.71	1.08
59% steel slag mixing	2653.58	2688.61	1.32
78% steel slag mixing	2632.45	2677.99	1.73
100% steel slag mixing	2654.23	2712.89	2.21

**Table 6:** The volume expansion of the mixture in its natural state when 6% cement was added.

Type of Mix	Initial Volume (cm^3^)	Volume after 7 Days (cm^3^)	Volume Expansion Rate (%)
0% steel slag mixing	2642.98	2647.72	0.18
21% steel slag mixing	2679.86	2692.46	0.47
40% steel slag mixing	2654.76	2681.23	1.00
59% steel slag mixing	2657.89	2691.24	1.25
78% steel slag mixing	2627.67	2671.89	1.68
100% steel slag mixing	2662.12	2711.12	1.84

**Table 7:** The swelling rate of the mixture when mixing 5% cement.

Immersion Time (days)	21% Steel Slag	40% Steel Slag	59% Steel Slag	78% Steel Slag	100% Steel Slag
1	0.11	0.22	0.37	0.46	0.54
2	0.33	0.39	0.52	0.70	0.77
3	0.41	0.48	0.70	0.75	0.93
4	0.53	0.53	0.86	0.87	1.16
5	0.61	0.69	0.93	1.06	1.32
6	0.66	0.74	1.15	1.29	1.57
7	0.79	0.85	1.33	1.41	1.73
8	0.86	0.99	1.51	1.63	1.87
9	0.93	1.03	1.64	1.82	2.29
10	1.07	1.21	1.73	2.11	2.67

**Table 8:** The swelling rate of the mixture when mixing 6% cement.

Immersion Time (days)	21% Steel Slag	40% Steel Slag	59% Steel Slag	78% Steel Slag	100% Steel Slag
1	0.08	0.12	0.27	0.36	0.44
2	0.24	0.27	0.41	0.57	0.74
3	0.32	0.45	0.65	0.72	0.88
4	0.47	0.52	0.83	0.83	1.08
5	0.56	0.66	0.92	0.97	1.25
6	0.61	0.70	1.09	1.16	1.52
7	0.67	0.81	1.31	1.35	1.67
8	0.70	0.93	1.52	1.57	1.81
9	0.82	1.01	1.59	1.74	2.11
10	0.97	1.11	1.61	1.91	2.47

**Table 9:** Training sample data.

Serial Number	Steel Slag Expansion Rate	Steel Slag Mixing	Specimen Expansion Rate	Serial Number	Steel Slag Expansion Rate	Steel Slag Mixing	Specimen Expansion Rate
1	0.11	0.21	0.11	26	0.73	0.59	1.64
2	0.23	0.21	0.41	27	0.82	0.59	1.73
3	0.28	0.21	0.52	28	0.11	0.78	0.46
4	0.34	0.21	0.61	29	0.15	0.78	0.69
5	0.44	0.21	0.66	30	0.23	0.78	0.74
6	0.58	0.21	0.79	31	0.28	0.78	0.87
7	0.64	0.21	0.86	32	0.34	0.78	1.06
8	0.73	0.21	0.93	33	0.44	0.78	1.29
9	0.82	0.21	1.07	34	0.58	0.78	1.41
10	0.11	0.4	0.22	35	0.73	0.78	1.82
11	0.15	0.4	0.38	36	0.82	0.78	2.11
12	0.23	0.4	0.47	37	0.11	1	0.54
13	0.34	0.4	0.69	38	0.15	1	0.77
14	0.44	0.4	0.74	39	0.23	1	0.92
15	0.58	0.4	0.85	40	0.28	1	1.16
16	0.64	0.4	0.99	41	0.34	1	1.32
17	0.73	0.4	1.03	42	0.44	1	1.57
18	0.82	0.4	1.21	43	0.58	1	1.73
19	0.11	0.59	0.37	44	0.64	1	1.87
20	0.15	0.59	0.51	45	0.73	1	2.29
21	0.23	0.59	0.69	46	0.15	0.21	0.33
22	0.28	0.59	0.85	47	0.28	0.4	0.52
23	0.34	0.59	0.93	48	0.44	0.59	1.15
24	0.58	0.59	1.33	49	0.64	0.78	1.63
25	0.64	0.59	1.51	50	0.82	1	2.67

**Table 10:** Test sample data.

Number of Days	Steel Slag Expansion Rate	Steel Slag Admixture	Mix Expansion Rate
2	0.15	0.21	0.33
4	0.28	0.4	0.52
6	0.44	0.59	1.15
8	0.64	0.78	1.63
10	0.82	1	2.67

**Table 11:** Prediction results output.

Measured Value	BP Projected Value	BP Inaccuracies
0.33	0.27141	−0.058592
0.52	0.54873	0.02873
1.15	1.1212	−0.028766
1.63	1.6101	−0.019886
2.67	2.7533	0.083275

**Table 12:** Indicators of projected results.

Cement Mixing	MAE	MSE	RMSE	MAPE
5% cement	0.043850	0.0024832	0.049832	6.0241%
6% cement	0.029548	0.0010899	0.033014	5.6820%

## Data Availability

All the data are available within the manuscript.
